# A Microwave Thermostatic Reactor for Processing Liquid Materials Based on a Heat-Exchanger

**DOI:** 10.3390/ma10101160

**Published:** 2017-10-08

**Authors:** Yongqiang Zhou, Chun Zhang, Tian Xie, Tao Hong, Huacheng Zhu, Yang Yang, Changjun Liu, Kama Huang

**Affiliations:** 1 College of Electronic and Information Engineering, Sichuan University, Chengdu 610065, China; Zhouyq1217@163.com (Y.Z.); chun-199012@163.com (C.Z.); yyang@scu.edu.cn (Y.Y.); cjliu@scu.edu.cn (C.L.); kmhuang126@126.com (K.H.); 2 State Key Laboratory of Efficient Utilization for Low Grade Phosphate Rock and Its Associated Resources, Wengfu Group, Guiyang 550014, China; chemostar@163.com; 3 School of Electronic Information Engineering, China West Normal University, Nanchong 637002, China; scu_mandela@163.com

**Keywords:** microwaves, liquid materials, PLC, thermostatic reactor, non-polar coolant, heat-exchanger

## Abstract

Microwaves have been widely used in the treatment of different materials. However, the existing adjustable power thermostatic reactors cannot be used to analyze materials characteristics under microwave effects. In this paper, a microwave thermostatic chemical reactor for processing liquid materials is proposed, by controlling the velocity of coolant based on PLC (programmable logic controller) in different liquid under different constant electric field intensity. A nonpolar coolant (Polydimethylsiloxane), which is completely microwave transparent, is employed to cool the liquid materials. Experiments are performed to measure the liquid temperature using optical fibers, the results show that the precision of temperature control is at the range of ±0.5 °C. Compared with the adjustable power thermostatic control system, the effect of electric field changes on material properties are avoided and it also can be used to detect the properties of liquid materials and special microwave effects.

## 1. Introduction 

Microwaves, as a clean and high effective energy, show many advantages over conventional heating methods [1,2,3,4,5,6]. The primary reason is that microwave heating mechanism, so called dielectric heating, is quite diffident from conventional heating. One feature of this process is volumetric heating, microwaves propagate deeply into materials (not always but only in microwave absorbing materials [7,8,9]) and thus simultaneously generate heat on both internal and external regions of the substance. Another feature is selective heating [10,11], microwaves preferentially heat materials with a large dielectric loss. Together, these mechanisms allow shorter heating durations and can carry out chemical reactions more efficiently [12]. Microwave heating can be widely found in industrial applications [13], Ma et al. reported that Ionic liquid-based microwave-assisted extraction (ILMAE) has been successfully applied in extracting essential oil and four kinds of biphenyl cyclooctene lignans from Schisandra chinensis Baill, dealing with a 25.0 g sample at 385 W irradiation power. The extraction time was shorted to 40 min and the solid–liquid ratio reached 1:12 [14]. Leadbeater et al. reported that the preparation of biodiesel using microwave processing can result in a fast, easy route to this valuable biofuel [15]. Rosi L et al. confirmed that the biodiesel yielding from waste cooking oil can be improved by using microwave [16,17]. The applications of microwave energy in treatment of materials show tremendous prospects and potential. However, the mechanism of promotion or influence on reactions by microwaves is still unclear.

Therefore, many researchers have studied the influences on reaction kinetics caused by microwave irradiation. John H. Booske et al. reported that strong microwave electric fields induce a (previously unknown) nonlinear driving force for (ionic) mass transport near surfaces and structural interfaces (e.g., grain boundaries) in ceramic materials. This driving force can influence reaction kinetics by enhancing mass transport rates in heterogeneous solid-state reactions [18]. Haoran Sun et al. found that the application of a high intensity microwave can affect and alter the intermolecular interaction in DMSO–methanol/ethanol systems, thereby leading to macroscopic dielectric property changes in mixtures [19]. In order to study the microwave effects on chemical reaction, the influences on reaction properties and material characteristics caused by electric field intensity need to be studied first. However, the reactor for detecting the influences of different constant electric field intensity on reactions and materials at the same temperature has not been studied yet. At present, the common temperature control strategy is to regulate the microwave irradiation power [20,21]. According to Sun’s experiment, the electric field intensity will influence the dielectric properties of materials. Therefore, regulating the microwave irradiation power to detect microwave special effects on materials is not applicable, especially in the verification of microwave non-thermal effects, which are still the focus of academic debate in practice [22,23,24,25,26,27]. The thermostatic control under different constant electric field intensity is also the crux of this problem. Hence, the fundamental work in testing the microwave special effects is to design a thermostatic reactor under different electric field intensity (microwave irradiation power). 

The objective of the study is therefore to develop a thermostatic reactor for studying the effects of electric field intensity on materials and reactions. For this purpose, we fabricated a reaction vessel for liquid samples, in which the microwave transparent coolant flows through the spiral heat-exchanger to adjust temperature. The liquid samples are irradiated by microwaves in a microwave multimode cavity (2.45 GHz) [28]. Meanwhile, a control system of constant temperature for processing liquid materials is employed, including a PLC and PID (proportional integral derivative) controller [29,30,31]. The thermostatic control system is built and different liquid samples are tested to achieve the stable temperature. The results indicate that the thermostatic reactor based on a heat-exchanger can control temperature accurately, the deviation between the controlling temperature and the target temperature is less than 0.5 °C under different electric field intensity. In addition, the reactor can be used to process the liquid materials at constant temperature under different electric field intensity. Furthermore, the reactor can also be used for detecting the effects of electric field on reactions and the microwave non-thermal effects.

## 2. Methodology 

### 2.1. Microwave Reactor with Heat-Exchanger

**Figure 1:**
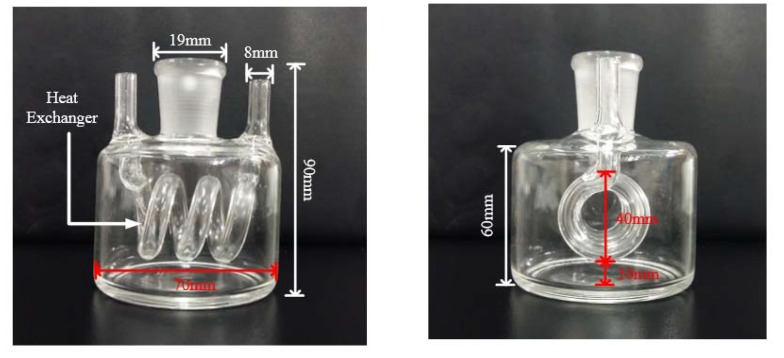
The proposed reactor with a heat-exchanger.

The special designed reaction vessel consists of a quartz bottle (i.d.: $68\times 10^{-3\;}m$ and o.d.: $70\times 10^{-3}\;m$ and h:$\;60\times 10^{-3}\;m$) for the liquid samples and a spiral tube (i.d.:$\;7\times 10^{-3}\;m$ and o.d.:$\;8\times 10^{-3}\;m$) for the coolant. In this paper, water, methanol, and ethanol are regarded as the liquid samples in the experiments. As a coolant, the Polydimethylsiloxane is completely microwave transparent. Meanwhile, the spiral tube is connected to a coolant tank which was placed in a freezer. The temperature inside the freezer is set to −60 °C. The schematic of the proposed chemical reactor is shown in Figure 1.

### 2.2. Experimental Setup 

**Figure 2:**
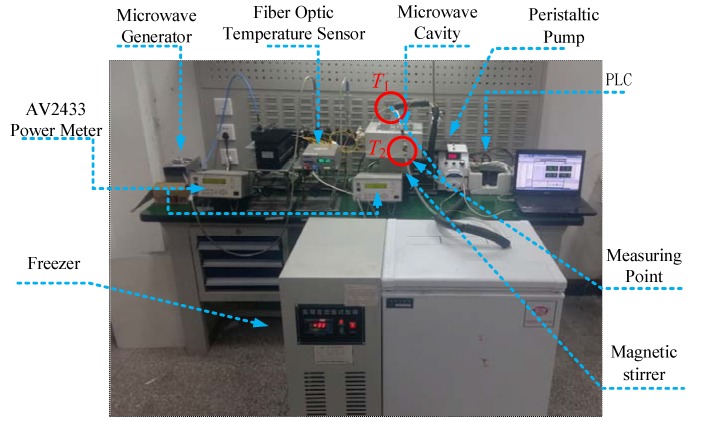
The photograph of the experimental system, *T_1_* is the temperature of liquid materials in reactor, *T*_2_ is the temperature of coolant flowing out from the heat-exchanger.

**Figure 3:**
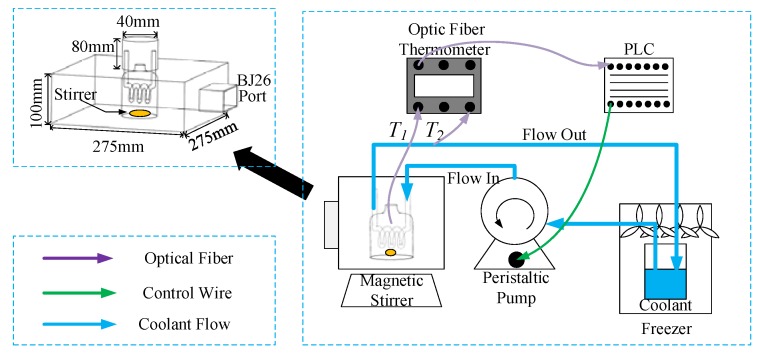
The local detail of the experimental setup.

The experimental system is set up as shown in Figure 2. The main waveguide is connected to an adjustable solid state microwave generator (adjustable power at 0~200 W, 2.45 GHz). Microwaves are orderly transmitted along the coaxial cable, the circulator and the directional coupler, then fed into the microwave cavity. The input power and the reflect power are measured by a microwave power meter (AV2433, the 41st Institute of China Electronic Technology Group Corporation, Qingdao, Shandong Province, China). As shown in Figure 3, the stirrer is used to mix the liquid, in addition, microwave transparent coolant flows through the silicone tube with the diameter of 8 mm, which is cooled by a freezer (DW-60 Cryogenic Freezer, Tianjin Zhongluda Instrument Technology Co., Ltd, Tianjin, China) and pumped to the heat-exchanger by the peristaltic pump (YZ1515X, Nanjing Runze Fluid Control Equipment Co., Ltd., Nanjing, China). The temperatures of the liquid samples in the reactor (*T_1_*) and the coolant flowing out from the heat-exchanger (*T_2_*) are measured by fiber optical temperature sensor (Reflex™ Signal Conditioner, Neoptix, Inc., Quebec City, Quebec Province, Canada), and the real-time temperture data are transmitted to the PLC control system for PID algorithm operation. The PID controller will change the coolant velocity by regulating the rotate speed of the peristaltic pump.

### 2.3. PID Controller

In this paper, the PID controller is employed to adjust the rotate speed of the peristaltic pump. With its three-term functionality (proportional controller, integral controller, and derivative controller) covering treatment to both transient and steady-state responses, the PID controller offers the simplest and most efficient solution for many control problems [29]. The differential equation of the PID regulator can be expressed by

(1) $$u(t)=Kp[e(t)+\frac{1}{Ti}\int_{0}^{t}e(t)dt+\frac{Td}{dt}de(t)]$$

where *Kp* is the proportional gain, *Ti* is the intergral time constant, and *Td* is the differential time constant. The transfer function of a standard PID controller is generally written in the ‘parallel form’ given by Equation (2) or the ‘ideal form’ given by Equation (3)

(2) $$G(s)=\frac{U(s)}{E(s)}=Kp(1+\frac{1}{Ti\cdot s}+Td\cdot s)$$

(3) $$=Kp+\frac{Ki}{s}+Kd\cdot s$$

where the *Ki* is the integral gain, and *Kd* is the differential gain. Theoretical analysis and control experiments show that a process of the conventional heating is a first order plus dead time (FOPDT) system [32,33,34], which is a highly dynamic system. The transfer function of the FODPT system is

(4) $$G(s)=\frac{K}{ts+1}e^{-\tau s}$$

where *K* is the system gain, *t* is the time constant, and *τ* is the dead time parameter. Based on FOPDT system, the common strategy for PID controller parameters tuning is the Ziegler–Nichols tuning method, and it is easy to systematically identify the controlled object [31,35,36,37].

## 3. Results and Discussion

### 3.1. Parameter Tuning

**Figure 4:**
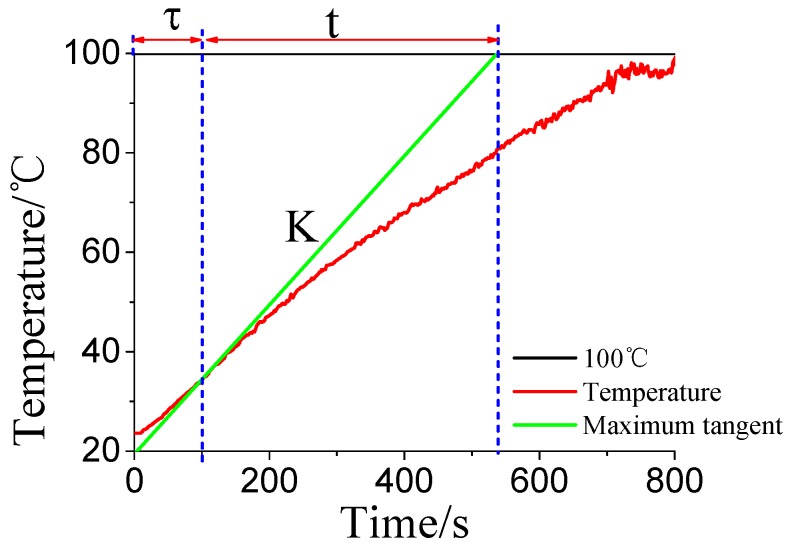
The curve of the system identification.

In this paper, Ziegler–Nichols tuning mehod is used to tune the PID parameters based on MATLAB program [38,39,40]. Figure 4 shows the temperature rise curve of water under 100 W microwave power and the system characteristic. The system gain *K* is the maximum tangent slope of the temperature rise curve, and intersection of the maximum slope tangent and the characteristic curve is dead time *τ*. The rest of time is the time constant *t*.

Acording to Equation (4), these parameters (*K*, *τ*, and *t*) can be easily calculated, in which *K* = 0.15, *τ* = 100 s and *t* = 438 s. So the system transfer function is

(5) $$G(s)=\frac{0.15}{438s+1}e^{-100s}$$

furthermore, the theoretical parameters of PID controller can be obtained as

(6) Kp=1.2TKτ=35.04

(7) Ti=2τ=200

(8) Td=τ2=50

**Figure 5:**
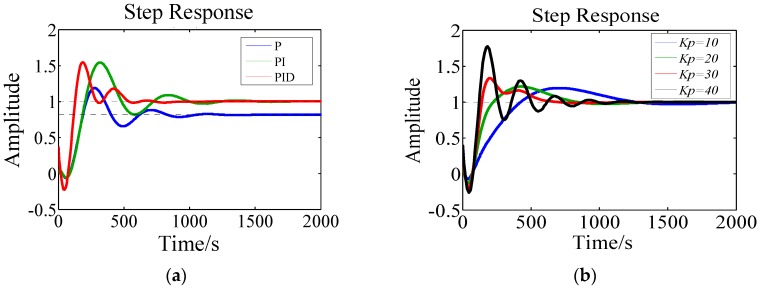

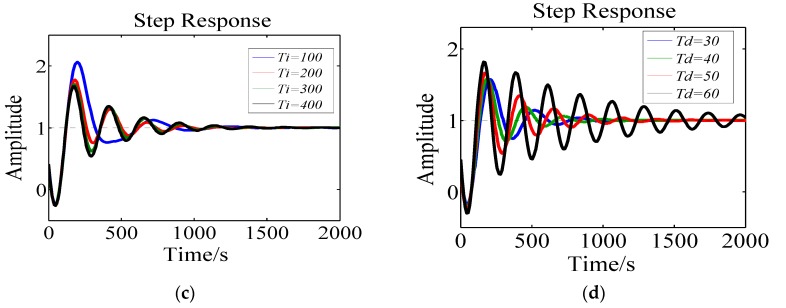
The curve of the system simulation result: (**a**) the simulation results of the P, PI, PID controller; (**b**) sweep results of *Kp*; (**c**) sweep results of *Ti*; (**d**) sweep results of *Td*.

According to the obtained values for system transfer function and system parameters, the PID controller is simulated, which are shown in Figure 5. Compared with P and PI controller, the PID controller is more stable and responds faster, as can be seen in Figure 5a. Then the paramaters of *Kp*, *Ti* and *Td* are swept respectively and the results are shown in the Figure 5b–d. The intergral time constant *Ti* and the differential time constant *Td* (from 30 to 50) have no obvious influence on the stability of the system, and the stability and overshoot of the system is primarily dependent on system gain *Kp* . Based on the simulation results, the initial paramaters of PID controller are given as: *Kp* = 30, *Ti* = 200 and *Td* = 50. 

Since the FOPDT system is a nonlinear system, which is sophisticated and difficult to predict, the oscillatory and overshoot of this system is different under different microwave power and control targets of temperature. In this case, for different microwave irradiation power and temperature targets, the multi-parameters of PID controller program are employed in this control system. The parameters of PID controller for different microwave power are shown in Table 1.

### 3.2. Temperature Control for Water

**Figure 6:**
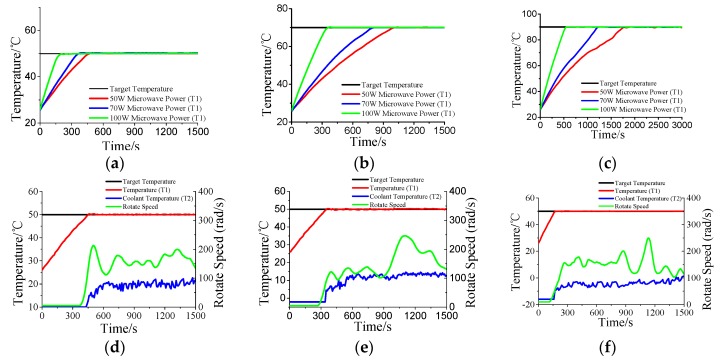
The temperature of water under different microwave irradiation power: (**a**–**c**) control temperature under 50, 70, and 100 W; (**d**–**f**) the temperature was controlled to 50 °C under 50, 70, and 100 W.

The experimental results of thermostatic control of water under the different microwave power are shown in Figure 6, in which a­-c show the results of temperature controlled under 50, 70, and 100 W microwave power. It can be seen that the temperature curve is basically consistent with the target value, and the control method is stable and reliable. The factors influencing the control effect are shown in Figure 6d–f, which illustrate that the rotate speed of pump is positively correlation with the temperature variations of water. Experiment shows that the coolant temperature and the flow velocity are the key factors influencing the constant temperature control. The influence on pump’s rotate speed by the coolant temperature can also be observed. In this experiment, the inflow temperature is rising because of circulating utilization of coolant, thus, the heat exchange velocity need to be increased through speeding up the rotate of pump. As shown in Figure 6d–f, with the increase in coolant temperature, the speed of the peristaltic pump also begins to accelerate. Additionally, the temperature rises more rapidly in the case of a high microwave power, which means the coolant temperature needs to be cooled to less than 0 °C as shown in Figure 6e,f. Furthermore, the coolant temperature is cooled to less than −10 °C in the case of 100 W and the rotate speed is relatively stable as compared to 70 W. This further demonstrates that the aid of the low temperature coolant improves the stability of the control system in the case of high microwave irradiation power. 

### 3.3. Temperature Control for Other Liquid

**Figure 7:**
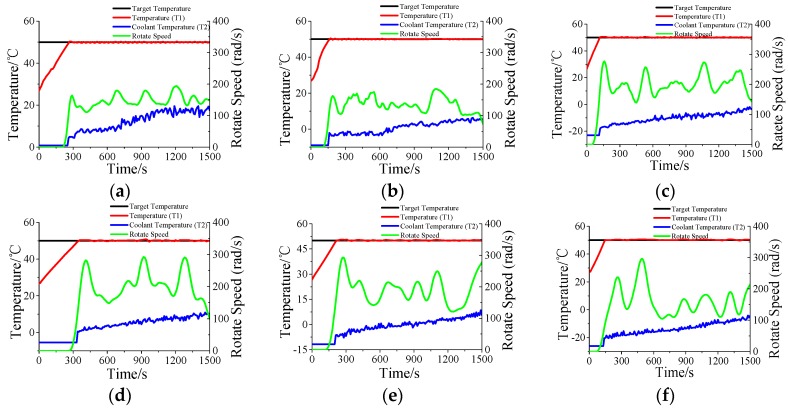
The temperature of other liquids at different microwave irradiation power: (**a**–**c**) the temperature of ethanol was controlled to 50 °C under 50, 70, and 100 W; (**d**–**f**) the temperature of methanol was controlled to 50 °C under 50, 70, and 100 W.

In addition to water, two other typical solutions (methanol and ethanol) are also used to test the constant temperature control effect of this system. Since these two solutions have a lower boiling point (64.5 °C and 78 °C), for this case, the target temperature is set as 50 °C. The experimental results are shown in Figure 7. From Figure 7, one can conclude that the system can be successfully used for the constant temperature control of methanol and ethanol solution. However, the coolant temperature must be cooled to less than 0 °C. The primary reason is that the specific heat capacities of methanol and ethanol are smaller than water. The temperature rise velocity is much higher than water under the same microwave power, which requires a much faster heat exchange velocity. Meanwhile, the rotate speed of the pump is higher than water under the same control temperature.

## 4. Conclusions

A microwave chemical reactor for processing the liquid materials under the same temperature but different microwave powers has been proposed. For hardware designing, PLC is employed to control the temperature and coolant velocity. In addition, for software designing, PID control algorithm is used for stabilizing the behavior and improving the precision of this system. Most of all, experiments are conducted to verify the accuracy of the control system. Several conclusions can be drawn from our research:

(1) A reactor for processing the liquid materials under the same temperature but different microwave power are designed.
(2) Different materials are used to test the control performance of the reactor, indicating that it can control the system temperature accurately.
(3) The reactor can be used to detect the special effect of electric field or microwaves on material properties, moreover, it will be helpful to detect the reaction kinetics parameters of the systems under microwaves.

## Figures and Tables

**Table 1:** The PID detail parameters for different irradiation power.

	Parameters	*Kp*	*Ti*	*Td*
Power/W				
50		70	650	185
70		42	340	85
100		30	200	50
